# A Comparative Policy Review of Tuberculosis Infection Control Policies Across Six Countries: Insights on Screening, Prevention, and Treatment Strategies

**DOI:** 10.1002/hsr2.72728

**Published:** 2026-07-06

**Authors:** Mohammad Veysi Sheikhrobat, Sajad Alizadeh, Kamal Shahamiri, Saeed Shahriari, Ahmad Tahmasebi‐Ghorrabi

**Affiliations:** ^1^ Department of Public Health Shoushtar Faculty of Medical Sciences Shoushtar Iran; ^2^ Khatam Al‐Anbia Hospital Shoushtar Faculty of Medical Sciences Shoushtar Iran; ^3^ Golestan Hospital Ahvaz Jundishapur University of Medical Sciences Ahvaz Iran; ^4^ Department of Endocrinology, Loghman Hakim Hospital Shahid Beheshti University of Medical Sciences Tehran Iran; ^5^ Ahvaz Jundishapur University of Medical Sciences Ahvaz Iran

**Keywords:** comparative policy analysis, global health governance, prevention, screening, treatment, tuberculosis

## Abstract

**Background and Aims:**

Tuberculosis (TB) infection remains one of the major global public health challenges, disproportionately affecting vulnerable and marginalized populations. This study aims to compare prevention, screening, and treatment policies for TB in selected countries to identify effective strategies and policy gaps that may inform national and international TB control programs.

**Methods:**

This comparative review analyzed the prevention, screening, and treatment policies of TB in Iran, the United States, Ireland, Japan, the United Kingdom, and Türkiye in 2023. Comprehensive searches were conducted in electronic databases (PubMed, Scopus, Web of Science, and Embase), as well as on the official websites of ministries of health and national TB programs in these countries. The inclusion criteria were the availability of policy documents addressing prevention, screening, and treatment, detailed information on implementation processes, and publication between 1993 and 2023. Data were extracted and synthesized based on the scope and structure of national TB strategies and alignment with WHO recommendations.

**Results:**

Findings indicated that the UK and the US have successfully implemented effective TB control policies through structured strategies, surveillance systems, and intersectoral coordination. Iran, Japan, and Türkiye achieved notable reductions in TB mortality by adopting similar evidence‐based policies. Although Ireland demonstrated a decline in incidence, its treatment success rate remained comparatively low. Challenges identified across countries included Multi‐Drug‐Resistant Tuberculosis (MDR‐TB), inequitable distribution of health services, limited financial and human resources, structural deficiencies in health systems, inadequate coordination of care, social stigma, and barriers related to migrant populations, which have collectively hindered TB control, especially in treatment.

**Conclusion:**

Effective TB control requires strengthening health system capacity, implementing integrated and active prevention programs, expanding screening among high‐risk and migrant populations, improving surveillance systems, and preventing drug‐resistant TB. Adequate financial investment, trained human resources, and coordinated policy implementation remain essential for sustainable TB control.

**Abbreviations**
DOTS: directly observed treatment, short‐course
GDP: gross domestic product
HIV: Human Immunodeficiency Virus
JATA: Japan Anti‐Tuberculosis Association
LTBI: latent tuberculosis infection
MDR‐TB: multi‐drug‐resistant tuberculosis
MMR: mass miniature radiography
SDGs: sustainable development goals
TB: tuberculosis
USPSTF: US Preventive Services Task Force
WHO: World Health Organization

## 1 Introduction

Tuberculosis is an airborne infectious disease caused by the bacterium *Mycobacterium tuberculosis*. The burden of TB remains a major public health concern worldwide and, following COVID‐19, it is among the leading infectious causes of death globally, exceeding mortality associated with Human Immunodeficiency Virus (HIV) in recent WHO reports [1, 2, 3]. The World Health Organization (WHO) recently reported 10.6 million new cases of infection and 1.3 million deaths in 2022 worldwide. According to WHO, its burden is not evenly distributed, as most cases occur in Southeast Asia (45%), Africa (23%), and the Western Pacific (18%). The Eastern Mediterranean (8.1%), the US (2.9%), and Europe (2.2%) reported a lower rate of TB cases in 2021 [4, 5]. Among the countries included in this comparison, TB incidence in 2022 ranged from 3 per 100,000 in the United States to 14 per 100,000 in Türkiye [6].

The World Bank estimates that TB causes a 4%–7% reduction in the gross domestic product (GDP) of some countries [7]. It reduces the labor force and productivity and limits a country's GDP [7]. Evidence suggests that poverty is involved in TB both on a macro scale and in individual and hierarchical analyses [8]. Additionally, TB affects indigenous populations as more than 95% of TB cases and deaths occur in low‐ and middle‐income countries (LMICs) [9]. Despite the availability of free drugs under the directly observed treatment, short‐course (DOTS) program, TB treatment is associated with high costs, exacerbating the economic uncertainty of households affected by this disease [10]. Nevertheless, since its introduction by the World Health Organization in the 1990s, the DOTS strategy has substantially contributed to global TB control through improved treatment adherence, higher treatment success rates, strengthened surveillance systems, and reductions in TB‐related mortality in many high‐burden settings [11]. Evidence suggests that the widespread implementation of DOTS has been associated with gradual declines in TB incidence and mortality worldwide [12].

WHO launched the “End of TB strategy within the framework of the United Nations Sustainable Development Goals (SDGs)” in 2014 [13]. The strategy aims to end TB by 2035 and achieve a 90% reduction in TB incidence from 100 cases per 100,000 in 2015 to 10 cases per 100,000 or less by 2035 [14]. The WHO's strategy to end TB has been developed in line with the SDGs [15]. Its burden has decreased gradually by 1.5%–2% per year during the past decade's [16]. However, key challenges, including MDR‐TB, delayed diagnosis, malnutrition, socioeconomic disparities, and health system challenges such as limited infrastructure, inadequate financing, workforce shortages, and fragmented service delivery, continue to hinder progress in reducing TB incidence [4, 17, 18].

Effective TB infection prevention and control (TB‐IPC) policies constitute a fundamental component of global TB control strategies [19]. These policies include administrative measures such as early identification and isolation of suspected TB cases, environmental interventions, including adequate ventilation and ultraviolet germicidal irradiation, and personal protective measures for healthcare workers and high‐risk populations [20, 21]. Evidence indicates that the implementation of comprehensive TB‐IPC policies in healthcare facilities, congregate settings, and community environments can substantially reduce transmission, improve early diagnosis, and strengthen treatment outcomes. In addition, integration of TB‐IPC measures within national health systems and primary healthcare services has contributed to reductions in TB incidence and mortality in several high‐ and middle‐income countries.

Despite advances in diagnostics, treatment, and prevention, TB remains a major public‐health problem worldwide [13, 15, 22]. A multi‐sectoral approach that encompasses universal access to care, community engagement, and integration with broader social policies is widely recommended to accelerate elimination. Recent evidence highlights the importance of global health governance, inter‐organizational collaboration, and policy coordination in strengthening TB control programs. Network‐based analyses have demonstrated that effective cooperation among international organizations, governments, and health institutions can improve TB policy implementation, resource allocation, and disease surveillance, particularly in developing countries [23, 24]. In addition, advances in evidence‐based treatment strategies, including shorter therapeutic regimens for drug‐resistant tuberculosis, have shown promising outcomes in improving treatment adherence and effectiveness [25]. Comparative analysis of TB policies across countries can identify transferable best practices for prevention and treatment [26, 27, 28].

Although previous studies have examined specific dimensions of TB control, such as treatment outcomes, surveillance systems, or organizational governance, comparative evidence integrating prevention, screening, and treatment policies across countries with diverse epidemiological and health‐system contexts remains limited. The present study contributes to the existing literature by providing a comprehensive cross‐national comparison of TB policy frameworks in six countries with differing healthcare capacities, governance structures, and TB burdens. By identifying common policy strengths, implementation gaps, and transferable strategies, this study offers practical insights for policymakers seeking to strengthen national and global TB control efforts.

## 2 Materials and Methods

### 2.1 Study Design and Review Framework

This study was conducted as a comparative policy review using a structured scoping review approach to examine TB prevention, screening, and treatment policies across selected countries. The methodological approach was informed by the scoping review framework proposed by Arksey and O'Malley and guided by the Preferred Reporting Items for Systematic Reviews and Meta‐Analyses (PRISMA) framework to ensure transparent identification, screening, eligibility assessment, and inclusion of relevant sources [29].

This comparative review analyzed the prevention, screening, and treatment policies of TB in Iran, the United States, Ireland, Japan, the United Kingdom, and Türkiye in 2023.

### 2.2 Selection of Countries

The selected countries (Iran, the United States, Ireland, Japan, the United Kingdom, and Türkiye) were purposively chosen to represent diverse epidemiological profiles, healthcare system structures, economic capacities, and TB control strategies. The selection also reflected variation in TB incidence, surveillance capacity, migration patterns, and implementation of WHO‐recommended TB policies, thereby enabling a meaningful comparative analysis across heterogeneous health‐system contexts.

### 2.3 Search Strategy and Data Sources

Comprehensive searches were conducted in electronic databases, including PubMed, Scopus, Web of Science, and Embase, as well as on the official websites of ministries of health and national TB programs in the selected countries.

The study selection strategy was additionally informed by the PICOS framework, including population (TB‐affected populations), interventions (prevention, screening, and treatment policies), comparisons (cross‐country policy approaches), outcomes (TB control indicators and policy effectiveness), and study types (policy documents, national guidelines, reports, and peer‐reviewed literature).

### 2.4 Eligibility Criteria and Study Selection

Eligibility criteria included: (1) official policy documents or scientific publications addressing TB prevention, screening, or treatment; (2) availability of implementation details or policy outcomes; (3) publication in English between 1993 and 2023; and (4) relevance to one of the selected countries. Editorials, opinion pieces, non‐systematic commentaries, and publications lacking sufficient methodological or policy information were excluded. Conference abstracts and working papers were not included in the final analysis because of limited methodological detail and concerns regarding consistency and peer‐review status.

A PRISMA‐based screening process was used to identify, screen, assess eligibility, and include relevant studies and policy documents. Duplicate records were removed before title and abstract screening. Full‐text assessments were subsequently conducted to determine final inclusion eligibility.

### 2.5 Data Extraction and Comparative Analysis

Data were extracted and synthesized based on the scope and structure of national TB strategies and alignment with WHO recommendations.

A standardized screening log was developed to ensure consistency in study identification, eligibility assessment, and data extraction across multiple evidence sources. This approach facilitated systematic comparison of heterogeneous policy documents and minimized selection inconsistencies during the review process.

Extracted data included information related to TB prevention strategies, screening policies, treatment protocols, governance structures, implementation mechanisms, surveillance systems, and alignment with WHO recommendations.

The categories used for comparative classification of TB policies in were developed based on key components of the WHO End TB Strategy [30, 31], including prevention, early diagnosis and screening, treatment and care, surveillance systems, health‐system support, and protection of vulnerable populations. These categories were selected because they represent core operational and policy dimensions commonly used in international TB control frameworks and national TB programs. The classification framework facilitated systematic comparison across heterogeneous health systems by enabling consistent evaluation of shared policy domains despite differences in epidemiological profiles, governance structures, healthcare financing, and service‐delivery models among the selected countries. The included sources were comparatively synthesized using thematic policy analysis across three predefined domains: prevention, screening, and treatment. All analyses were descriptive and comparative in nature, and no inferential statistical hypothesis testing was performed.

### 2.6 Quality Assessment

Methodological quality and potential risk of bias were assessed based on the clarity, comprehensiveness, methodological transparency, and source credibility of the included documents and publications. Official national guidelines, WHO reports, and peer‐reviewed articles were prioritized during evidence synthesis.

## 3 Results

### 3.1 Document Screening Results

Identification phase: In this study, 3156 records were identified through database searches (PubMed, Scopus, Web of Science, and Embase), and an additional 78 records were retrieved from other sources, including gray literature and official institutional websites.

Screening phase: After removing 751 duplicate records, 2405 records remained for title and abstract screening. Following the screening process, 178 records were retained for further evaluation.

Eligibility phase: A total of 42 full‐text articles were assessed for eligibility based on the predefined inclusion and exclusion criteria, methodological relevance, and availability of policy‐related information.

**FIGURE 1:**
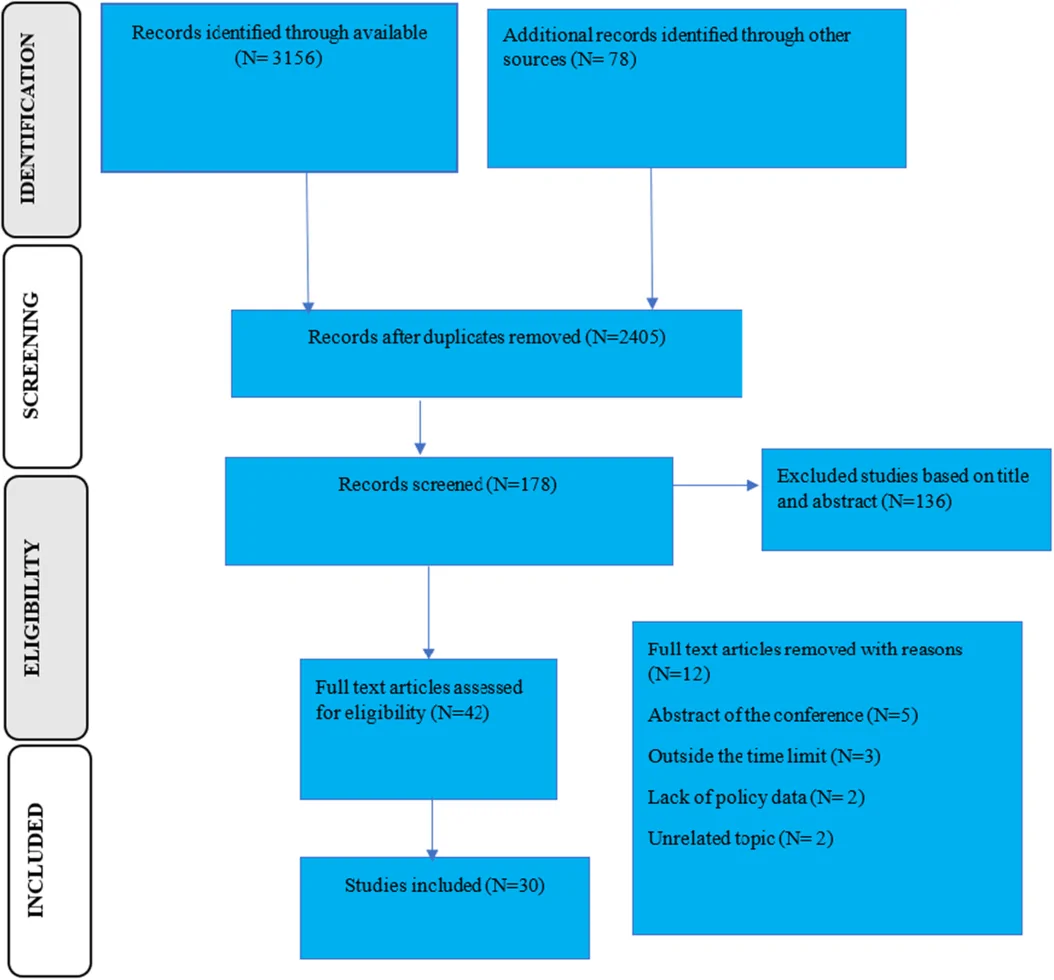
PRISMA flow chart for study selection.

Inclusion phase: Ultimately, 30 studies were included in the final comparative review and policy analysis (Figure 1). All included studies were published between 1993 and 2023.

In addition to scientific databases, gray literature was searched through the websites of the Ministries of Health of the selected countries, tuberculosis‐related websites, the World Bank, the World Health Organization, and Google, in order to access and review relevant policy documents. After examining Internet‐based sources, their primary characteristics were compared with those of the reviewed countries. The key findings from both scientific databases and Internet‐based sources are presented in Tables 1, 2, 3.

### 3.2 Screening

Evidence from comparative TB control studies suggests that early detection and systematic screening programs are essential for reducing TB transmission and improving treatment outcomes.

The UK demonstrated strong screening performance through tracing close contacts, screening programs in prisons and healthcare centers, and comprehensive national surveillance systems. In addition, individual risk assessments for healthcare personnel and targeted screening in specialized environments contributed substantially to early detection and improved TB control outcomes. These strategies contributed to high case‐detection rates and effective disease monitoring.

Iran has the lowest case‐detection rate (72%) among the studied countries and, after Türkiye, the highest TB incidence (11 per 100,000 population) (Table 1) [32]. Screening practices in Iran remain limited to basic methods, whereas comparator countries have adopted more advanced and targeted screening approaches for high‐risk populations (Table 3). Additional barriers include insufficient reporting of new cases, lack of standardized screening protocols, and limited access of suspected patients to healthcare facilities.

**TABLE 1:** Comparison of TB epidemiology and health expenditure in selected countries (2022).

		Ireland	UK	US	Japan	Türkiye	Iran
TB epidemiology	TB incidence (per 100,000 people) (2022)	5	8	3	10	14	11
	The rate of diagnosis of TB cases (2022)	86	92	87	87	83	72
	TB treatment success rate (percentage of new cases) (2021)	3	86	64	64	81	83
	Current health expenditure (% of GDP)	6.07	11.34	16.57	10.82	4.57	5.77
GINI Index		70	67.4	59.4	55.8	55.1	34.8
Total health expenditure per capita (2022 international dollars)		6045	5492	10,646	5250	1036	1082
Human Development Index (2022)		8	18	21	19	48	76
Life expectancy at birth (female/male)		80/83	79/83	76/80	81/86	76/80	75/79

Japan utilizes mass miniature radiography (MMR) and community‐based screening approaches to facilitate earlier diagnosis of TB cases. Despite these measures, delays in diagnosis and healthcare‐seeking behavior continue to affect TB control efforts [33, 34].

Ireland has implemented screening strategies aimed at reducing delays in diagnosis and improving rapid initiation of treatment. These strategies were strengthened through multidisciplinary workforce training and targeted screening approaches for vulnerable and high‐risk populations. Screening approaches focus particularly on vulnerable and high‐risk populations.

The United States implements annual screening of healthcare workers, latent TB screening programs, occupational risk assessments, and contact tracing systems integrated into occupational and public health services.

Türkiye utilizes passive contact tracing approaches by encouraging symptomatic individuals to seek medical consultation. However, limited healthcare access in rural and deprived areas and insufficient coordination between healthcare services continue to affect screening effectiveness.

### 3.3 Prevention

Comparative evidence from global TB policy evaluations indicates that countries with integrated prevention strategies, public awareness programs, and protection of high‐risk populations achieve better TB control outcomes.

The UK follows all programs and policies related to TB prevention and treatment (Table 2). In addition to strategies commonly implemented in the studied countries, the UK has adopted preventive treatment for people at risk, including individuals infected with HIV and close contacts of TB patients. Other preventive strategies include combating social and economic determinants such as poverty and poor nutritional conditions, screening in special environments such as prisons and healthcare facilities, school‐based screening programs, and implementation of a comprehensive national TB program (Table 3). These measures have contributed to high diagnosis and treatment success rates and a downward trend in TB mortality (Figure 3).

**TABLE 2:** Adopted policies related to the prevention and treatment of TB in selected countries.

Topic	Programs and policies	Ireland	US	UK	Japan	Türkiye	Iran
Background and context	National TB Policy Plan	Yes	Yes	Yes	Yes	Yes	Yes
	Availability of epidemiological data	Yes	Yes	Yes	Yes	Yes	Yes
Governance of the TB program	Structure, Human resources, and Funding	Yes	Yes	Yes	Yes	Yes	Yes
	Evidence‐based policy and data appropriate for action	Yes	Yes	Yes	Yes	Yes	Yes
TB/HIV, comorbidities, and priority populations	Knowledge and awareness of TB among the general population and high‐risk groups	No	Yes	Yes	Yes	Yes	No
	Screening of high‐risk groups	Yes	Yes	Yes	Yes	Yes	Yes
	TB treatment guidelines	Yes	Yes	Yes	Yes	Yes	Yes
	Harm reduction strategies	Yes	Yes	Yes	Yes	Yes	Yes
	Promotion of partnerships	Yes	Yes	Yes	Yes	Yes	Yes
	Resource mobilization	Yes	Yes	Yes	Yes	Yes	Yes
	Using the financial resources of international organizations	Yes	No	No	No	Yes	Yes
	Cooperation with international organizations	Yes	Yes	Yes	Yes	Yes	Yes
	Treatment with public funds	Yes	Yes	No	Yes	Yes	Yes
	National list of essential drugs or TB drugs subsidized by the government	Yes	Yes	No	Yes	Yes	Yes
	TB surveillance system	Yes	Yes	Yes	Yes	Yes	Yes
	Harm reduction policy for vulnerable groups	Yes	Yes	Yes	Yes	Yes	Yes
TB prevention		Yes	Yes	Yes	Yes	Yes	Yes
TB diagnostics		Yes	Yes	Yes	Yes	Yes	Yes
TB treatment		Yes	Yes	Yes	Yes	Yes	Yes

**TABLE 3:** Adopted strategies of prevention, screening, and treatment related to TB infection in selected countries.

	Ireland	UK	US	Japan	Türkiye	Iran
Prevention	− Limited BCG vaccination for children at risk − Public awareness and education (targeted high‐risk groups and health‐care settings) − Preventive treatment of latent TB − Strengthening human resources by training multidisciplinary forces for effective prevention of TB − Paying attention to the social factors affecting TB − Strengthening the prevention of TB in high‐risk groups [35, 36, 37]	− BCG vaccination − Preventive treatment for people at risk, such as people with HIV or in contact with a patient with TB − Education and information − Research and development of vaccines and new drugs − Fighting social and economic factors (eliminating poverty and improving nutritional conditions) [38, 39, 40, 41]	− Limited use (in high‐risk people) of BCG vaccination − Annual TB training: Providing annual TB training for all healthcare personnel − Annual TB screening of healthcare personnel − Research and development of vaccines and new drugs [42, 43]	− BCG vaccination − Determining TB prevention week (September 24–30) to increase awareness and provide accurate information − Establishment of the Japan Anti‐TB Association (JATA) for education, research, and promotion of public concern about TB prevention − Research and development of vaccines and new drugs [44, 45, 46]	− BCG vaccination for infants − Public awareness campaigns on TB prevention and control measures − Mandatory use of personal protective equipment (PPE) by healthcare staff − Integration with other health services, such as AIDS/TB collaborative activities − Screening of people with latent TB [47, 48, 49, 50, 51]	− BCG Vaccination − Education and information about the disease on the National Day to Fight TB − Training in schools by healthcare experts [52, 53]
Screening	− Screening for the time needed to diagnose and start appropriate treatment for patients with TB − Targeted screening of groups at risk, such as immigrants from high‐prevalence countries, prisoners, homeless people, people with AIDS, and so forth. − Test (TST) − QuantiFERON‐TB Gold Plus blood test − Chest X‐ray imaging for active diagnosis of TB [42, 43, 44]	− Targeted screening for high‐risk groups (such as immigrants from high‐prevalence countries, homeless people, and drug users) − Performing TST and QuantiFERON‐TB Gold tests − Screening in special environments (such as prisons and healthcare centers) − Tracing close calls − Rapid molecular diagnostic tests (PCR) − Screening programs in schools − Rapid molecular diagnostic tests − Screening programs in schools [54, 55, 56, 57, 58]	− Screening of close contacts of patients with contagious TB to identify people with latent TB − Individual risk assessment of all healthcare staff exposed to TB infection − Screening of people without previous TB using (IGRA) or (TST) − Performing targeted screening: focusing on high‐risk populations [43, 59, 60, 61, 62]	− Mass miniature radiography (MMR) to screen patients − Pre‐entry TB screening for citizens of countries with high prevalence (including chest radiography and tuberculin skin test or interferon‐gamma release test (IGRA)) [45, 63, 64]	− Chest X‐ray as a primary screening tool − Microscopic sputum smear − Active contact tracing for confirmed cases to identify and screen suspicious cases − Passive contact tracing by encouraging people with symptoms to visit the physician − Performing molecular tests such as Xpert MTB/RIF assay for rapid diagnosis of TB and resistance to rifampicin [65]	− Chest X‐ray − Tuberculin skin test − Direct microscopic smear of sputum [52]
Treatment	− National program to fight TB (2024–2030) − DOTS strategy − Integration with other health services, such as the AIDS program − Supporting vulnerable groups [36, 37]	− US National TB Program [56] − DOTS strategy − Drug‐resistant TB treatment (MDR‐TB and XDR‐TB) [41, 66]	− Direct observation treatment (DOT): − 6‐month or 9‐month RIPE standard regimen − New 4‐month regimen [67]	− Japanese‐model DOTS strategy − Japan National TB Program − Hospital and community‐based treatments − Treatment of drug‐resistant TB (MDR‐TB and XDR‐TB) [45, 68]	− National TB Control Program − DOTS strategy − Treatment of drug‐resistant TB (MDR‐TB and XDR‐TB) [65]	− National program to fight TB − DOTSII strategy or stop TB strategy − Treatment of drug‐resistant TB (MDR‐TB and XDR‐TB) [52]

Iran has adopted most prevention strategies implemented in the comparator countries; however, initiatives aimed at improving TB knowledge and awareness among the general population and high‐risk groups remain limited (Table 2). Preventive activities are largely restricted to BCG vaccination and school‐based education programs. Broader groups, including healthcare workers, vulnerable workers, and the general population, receive limited systematic TB education.

Japan has implemented nearly all major prevention strategies adopted by the comparator countries, except for the use of international financial resources for TB control (Table 2). Distinctive preventive measures include TB Prevention Week (September 24–30), educational and research activities through the Japan Anti‐TB Association (JATA), and community‐based prevention initiatives.

Ireland follows most TB prevention policies implemented in the comparator countries and has strengthened prevention through training multidisciplinary healthcare personnel, identifying social determinants affecting TB transmission, and focusing preventive interventions on high‐risk populations (Table 3). However, similar to Iran, public awareness initiatives targeting the general population remain limited (Table 2).

The United States has implemented a broad range of preventive policies, including annual screening of healthcare personnel, contact tracing for latent TB infection (LTBI), and risk assessment programs for healthcare staff exposed to TB. The US has largely discontinued routine BCG vaccination and instead emphasizes targeted testing and treatment of LTBI.

Türkiye has implemented most prevention policies observed in the comparator countries (Table 2). Distinctive preventive measures include mandatory use of personal protective equipment by healthcare workers and integration of TB prevention with primary healthcare services and collaborative AIDS/TB activities.

Across all countries, persistent preventive challenges include health inequalities, stigma, vulnerable migrant populations, and socioeconomic barriers affecting access to preventive services.

### 3.4 Treatment

Existing evidence indicates that successful TB treatment outcomes depend on integrated healthcare delivery systems, effective surveillance, access to medicines, and management of MDR‐TB.

The UK has the highest rates of diagnosis and treatment success among the studied countries (Table 1). Despite reductions in mortality rates and successful implementation of TB treatment programs, several challenges remain, including high TB rates in urban areas [69, 70], increased disease burden among foreign‐born populations [20], health inequalities uneven quality of care, lack of coordination in healthcare delivery, increasing complexity of MDR‐TB treatment [69], and the need for advanced surveillance systems to identify and manage MDR‐TB cases [70].

Although Iran achieved a treatment success rate of 83% in 2021, its relatively high TB incidence and low diagnosis rate indicate major gaps in early detection and treatment continuity. Iran's Gini index reflects relatively equitable wealth distribution compared with the selected countries; however, healthcare expenditure per capita and the share of health expenditure in gross national product remain low, comparable only to Türkiye (Table 1). Challenges affecting treatment outcomes include MDR‐TB, recurrent disease, poverty, homelessness, unequal access to healthcare services, geographic disparities in TB distribution, and TB/HIV co‐infection [71, 72, 73, 74]. Reduced financial resources for TB programs and fragmented healthcare coordination further complicate TB control [75].

**FIGURE 2:**
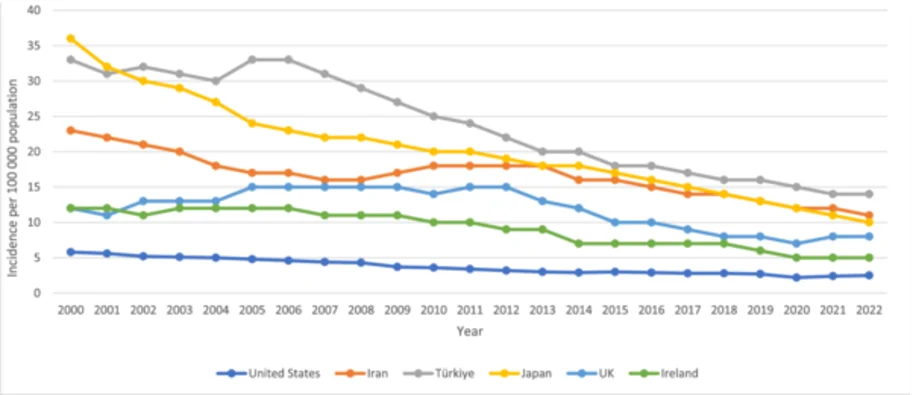
Trends of TB incidence (per 100,000 people) in selected countries between 2000 and 2022 [6].

**FIGURE 3:**
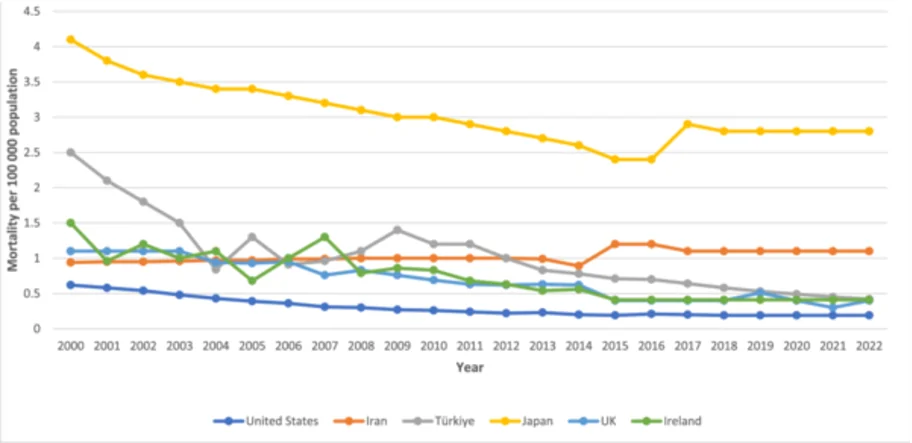
Trends of TB mortality rate (per 100,000 people) in selected countries between 2000 and 2022 [76].

Japan had the highest TB mortality rate among the studied countries in 2022 (2.8 per 100,000 population) (Figure 2). Although TB incidence has declined substantially since 2000, the current rate remains relatively high compared with other low‐incidence countries (Figure 3). Japan provides both hospital‐based and community‐based TB treatment services. Nevertheless, MDR‐TB, delays in diagnosis, and limitations within the public health system continue to challenge TB control efforts [33, 34, 77].

Ireland demonstrated low TB mortality and incidence rates; however, treatment success rates remained comparatively lower than in several comparator countries [32]. Ireland integrates TB treatment with HIV/AIDS programs and provides support services for vulnerable populations. Despite these strategies, TB incidence increased by 208 cases in 2023 compared with the previous year [78, 79]. Additional challenges include increasing TB burden among foreign‐born populations [79], incomplete reporting systems, lack of coordination between healthcare providers [78], health inequalities [78, 80], stigma, and increasing complexity of drug‐resistant TB treatment [79, 81].

The United States has the lowest TB incidence and mortality among the studied countries but a treatment success rate of 64%, lower than the UK and Iran (Table 1). The US utilizes DOTS for RIPE treatment regimens and maintains broad treatment and surveillance programs. However, the country does not use international financial resources for TB control, relying primarily on domestic funding. Challenges include inadequate training opportunities for healthcare providers, reduced funding, limited coordination between TB programs and private providers [82], interruptions in TB drug supply, social and structural barriers affecting vulnerable populations, and insufficient surveillance systems for detecting drug‐resistant TB cases [82, 83].

Türkiye has the highest TB incidence among the studied countries [69] despite a downward trend in TB‐related mortality over time (Figure 3). The National TB Program has improved treatment outcomes through integration with collaborative TB/AIDS services and conventional treatment strategies. Nevertheless, major challenges remain, including limited access to healthcare services in deprived areas, stigma and discrimination in healthcare utilization [84, 85], resource limitations, insufficient coordination between health and treatment services, and emerging MDR‐TB [85, 86].

## 4 Discussion

TB is a serious infectious disease and a major global health concern. Its prevention and treatment reduce TB transmission, infection rates, and mortality, improve quality of life, and decrease the economic burden on societies [87]. Effective TB prevention and control require comprehensive and innovative policies and programs. This study compared TB prevention, screening, and treatment policies across selected countries and provides valuable insights for policymakers and health‐system managers.

The present comparative analysis demonstrated substantial variation in TB prevention, screening, and treatment outcomes across the selected countries. The findings highlight important policy implications regarding the relationship between health‐system capacity, surveillance performance, and TB outcomes [88]. Countries with stronger healthcare investment and integrated surveillance systems, such as the UK and the United States, generally demonstrated lower TB incidence and more effective case detection and monitoring mechanisms. In contrast, countries with lower health expenditure and structural limitations in healthcare delivery, including Iran and Türkiye, faced greater challenges in early diagnosis, equitable access to services, and TB control performance. These findings emphasize the importance of sustained financial investment, strengthened surveillance infrastructure, targeted screening strategies, and integrated national TB policies to improve TB prevention and treatment outcomes across diverse epidemiological settings.

The findings highlight important policy implications regarding the relationship between health‐system capacity, surveillance performance, and TB outcomes [88]. Countries with stronger healthcare investment and integrated surveillance systems, such as the UK and the United States, generally demonstrated lower TB incidence and more effective case detection and monitoring mechanisms. The higher proportion of GDP expenditure allocated to health in these countries was associated with stronger surveillance infrastructure, broader screening coverage, and more coordinated TB control systems. In contrast, countries with lower health expenditure and structural limitations in healthcare delivery, including Iran and Türkiye, faced greater challenges in early diagnosis, equitable access to services, and TB control performance. These findings emphasize the importance of sustained financial investment, strengthened surveillance infrastructure, targeted screening strategies, and integrated national TB policies to improve TB prevention and treatment outcomes across diverse epidemiological settings.

Investigating TB prevention programs, policies, and strategies indicates that, in the area of prevention, approaches such as TB vaccination, education and awareness campaigns for patients, the general public, and healthcare providers, implementation of preventive measures in high‐risk settings, and research on new vaccines are commonly adopted across the studied countries. However, certain strategies are followed by the studied countries. There are also some strengths and weaknesses in similar strategies. Iran continues to use BCG vaccination as its primary strategy to prevent TB [89]. However, the US has mostly stopped routine BCG vaccination and focused on targeted testing and treatment of latent TB infection (LTBI) [90]. Japan also promotes BCG vaccination and community‐based prevention measures to prevent TB. The prevention system in Türkiye also focuses on contact tracing, preventive treatment, and the involvement of the primary care system based on the WHO guidelines [91].

The differences among countries regarding TB control are largely attributable to the facilitation, strengthening, and proper implementation of control strategies. Federal support and funding, strong public health infrastructure, partnership, and cooperation between different stakeholders and interaction with the community in the US [92, 93, 94], various types of TB, and focusing on health inequalities in the UK are some of the factors facilitating and strengthening TB control strategies in these countries [95, 96]. These factors were associated with the successful performance of these countries in preventing and controlling TB.

In the screening section, the results indicate that the studied countries follow almost similar strategies. The strategies of chest imaging, tuberculin skin test, direct microscopic smear of sputum, and active and passive contact tracing, are followed by all the studied countries. However, special strategies such as screening in specific environments, screening for the time required to diagnose and start appropriate treatment, targeted screening, and individual risk assessment of all healthcare staff exposed to TB infection in Ireland, the UK, and the US could make a difference in outcomes.

However, there are some challenges and barriers to the correct implementation of strategies in countries with poor performance, which have affected TB control in these countries. The study by Ebrahimoghli et al. revealed that screening programs for TB patients in Iran are inactive, focusing on those who were referred to healthcare centers and did not consider the high‐risk population and immigrants. However, the US Preventive Services Task Force (USPSTF) in the US recommends screening for LTBI in adults at higher risk, such as those who have lived in countries with a high burden of TB [59, 96]. In the UK, the focus has been on screening immigrants from high‐risk countries [97].

A study also revealed that there is no documented LTBI screening program for the general adult population in Iran, and it has focused more on the diagnosis and active treatment of TB cases than on LTBI screening for disease control [98]. Despite a relatively high treatment success rate among diagnosed patients (83%), Iran's low case‐detection rate (72%) and its higher incidence compared with most of the countries studied indicate persistent structural and programmatic challenges that continue to hinder effective TB control. Iran faces several barriers in identifying TB cases, including inactive or limited active case‐finding programs, insufficient community participation, lack of standardized screening protocols, restricted access of high‐risk groups and immigrants to healthcare services, limited awareness programs, and weaknesses in surveillance and reporting systems [99]. These findings suggest that acceptable treatment outcomes among diagnosed patients do not necessarily reflect effective early detection and comprehensive TB control at the population level. However, in Ireland, people at high risk of TB, such as those with weakened immune systems or those who have lived in countries with high TB prevalence, are targeted for screening [100].

In the present study, it was found that all studied countries follow the strategies of the national TB control program and the DOTS strategy in the treatment sector. Results revealed that Ireland had the worst performance in successfully treating new TB cases among the studied countries. The strategy of drug‐resistant TB treatment is not followed in Ireland. It was also found that the lack of plan and strategy for patients who developed MDR‐TB was associated with failure and ineffectiveness of TB treatment. Additionally, the strategies of integrating the TB treatment program with other health services, such as the AIDS program and supporting vulnerable groups failed to guarantee the success of TB treatment. It seems that written programs are not a guarantee for the success of the treatment and supporting measures and their correct implementation are vital.

Studies have revealed that non‐completion of the treatment course, defects in TB vaccination, unsuccessful treatment, non‐reporting of treatment results, old age of patients, unemployment, poverty, suppression of the immune system, anti‐TB MDR‐TB, a long period of being a carrier in the community, and pre‐hospital and in‐hospital delays are some of the reasons for Ireland's failure to diagnose and treat TB. Thus, Ireland has considered additional support for the elderly, unemployed, drug‐resistant patients, and patients with suppressed immune systems. Additionally, other strategies such as completion of treatment, appropriate screening, research to detect new cases of TB, and intervention to deal with delay in diagnosis and treatment have been adopted to improve the disease treatment process [56, 57]. However, the UK has been successful in treating patients. Studies revealed that factors such as advanced diagnostic and treatment capabilities [95], the implementation of the national TB surveillance system [41], and a focus on health inequalities [95, 96] were effective in strengthening and correctly implementing control strategies and policies in the treatment sector.

Results also revealed that the UK provides integrated care for co‐morbidities such as AIDS and diabetes in the healthcare system for better coordination of care for TB patients to succeed in the TB treatment [60]. Other studied countries also faced challenges in this sector that have affected the successful treatment of patients. Iran's national TB control program focuses on DOTS for drug‐susceptible TB, but it faces some challenges in managing of multi‐MDR‐TB cases. However, the US has stronger treatment guidelines and access to newer drugs for TB treatment, allowing for more effective management of drug‐resistant cases [90]. The study by Lee et al. also indicated that Japan follows the strategy of free TB treatment, including diagnostic tests and drugs for all patients regardless of their nationality or insurance status to succeed in TB treatment [101]. In general, challenges such as the higher incidence and prevalence, the susceptibility of patients to diabetes, the presence of MDR‐TB, the lack of proper implementation of the DOTS program, the lack of accurate and systematic monitoring of immigrants and their TB control programs and unfavorable outcomes of treatment in patients have been the reasons for the lack of success in the treatment of TB.

Finally, based on the comparison of strategies and policies with the consequences of TB control in selected countries, it can be said that adopting specific strategies, supporting strategies, continuing and following strategies, and identifying challenges and responding to them were accompanied by differences in the consequences of TB control in the studied countries. Despite a comprehensive search among several databases and gray literature sources, some limitations remained in the review. First, although many efforts were made to ensure that the search strategy and reviewed literature were extensive, some documents may be confidential and not publicly available. Thus, this review may not cover all relevant actions, considerations, and outcomes, leading to an incomplete picture of policies and programs related to TB prevention, screening, and treatment. Second, the countries were selected based on their performance and the existence of programs and policies, while other countries could also be examined for their different performance, programs, and policies. World Health Organization's End TB Strategy, which establishes ambitious targets for reducing TB incidence and mortality by 2035. Our analysis indicates that, although progress has been made, none of the countries included in this study has fully met the WHO targets. Accelerating the elimination of this disease requires sustained commitment, adequate funding, and stronger integration within health systems.

### 4.1 Policy Implications and Recommendations

This comparative analysis of TB prevention, screening, and treatment policies across various countries provides practical insights for policymakers at the global level. The following recommendations are derived from successful strategies implemented in diverse international contexts and emphasize evidence‐based, adaptable approaches aligned with the standards of the World Health Organization to strengthen global TB control efforts.

**Recommendation 1: Strengthening Integrated TB Case Detection, Screening, and Surveillance Strategies**

Targeted screening for latent TB among immigrants, healthcare personnel, and vulnerable populations should be prioritized. Utilizing advanced diagnostic tools such as Interferon‐Gamma Release Assays (IGRA) and integrating them into national guidelines—alongside community‐based outreach and multidisciplinary training programs—can enhance case detection rates, reduce disease transmission, and support global objectives for TB incidence reduction.

In addition, passive case reporting and passive case detection mechanisms should be strengthened within national surveillance systems to ensure timely identification, notification, and management of symptomatic individuals presenting to healthcare facilities. Integrating passive reporting into routine primary healthcare services may improve continuity of care, support comprehensive TB management, and enhance the completeness and accuracy of national TB surveillance data.

**Recommendation 2: Enhancing Preventive Measures Through Vaccination and Awareness Programs**

BCG vaccination should be supplemented with comprehensive public awareness campaigns aimed at combating social stigma and improving public knowledge. Expanding preventive programs to include prophylactic therapy for contacts of TB patients and multimedia‐based education for healthcare providers and high‐risk groups—supported by funding collaborations with non‐governmental organizations (NGOs)—can help reduce TB incidence through early intervention and equitable access across diverse populations.

**Recommendation 3: Improving Treatment Protocols for Drug‐Resistant TB and Developing Integrated Care Models**

DOTS and managing MDR‐TB through integrated service delivery—such as linkage with HIV programs—is essential. Developing national treatment guidelines for resistant cases, ensuring free access to essential medicines, and training healthcare personnel to deliver coordinated primary care can increase treatment success rates, limit the spread of resistance, and address comorbidities effectively.

**Recommendation 4: Promoting Multisectoral Collaboration to Overcome Structural Barriers**

Strengthening cooperation among ministries and social institutions to address social determinants of health—such as poverty and migration—along with increasing the share of health budget allocations, can promote equity in service delivery, enhance program sustainability, and improve the resilience of health systems across different epidemiological contexts.

**Recommendation 5: Investing in Surveillance and Research for Adaptive Policymaking**

Developing digital surveillance systems for real‐time monitoring of MDR‐TB and treatment outcomes, coupled with financial support for innovative research and periodic program evaluations, is strongly recommended. These actions foster dynamic, evidence‐based policymaking, reduce diagnostic delays, and strengthen global resilience against co‐infections and emerging health threats.

## 5 Conclusion

This comparative policy analysis of TB prevention, screening, and treatment strategies across Iran, the United States, the United Kingdom, Japan, Ireland, and Türkiye highlight substantial variations in program design, implementation, and outcomes. While countries such as the UK and Japan have demonstrated relative success through active screening, structured treatment protocols, and robust surveillance systems, challenges remain in nations where drug‐resistant TB management, sustainable financing, and comprehensive support for vulnerable groups are underdeveloped.

For Iran, the findings underscore the urgent need to strengthen active case detection among high‐risk populations, establish a national TB surveillance system, and adopt structured protocols for drug‐resistant TB treatment. Long‐term sustainability requires integrating TB services into primary health care, ensuring reliable financing mechanisms, and improving vaccination and monitoring coverage. Additionally, social protection for vulnerable populations and expanded investment in research and innovation are critical for long‐term resilience.

Overall, policy recommendations derived from this analysis emphasize evidence‐informed strategies that are feasible, context‐specific, and aligned with international best practices. Prioritizing these measures can enhance TB control in Iran and provide valuable lessons for other countries facing similar epidemiological and health system challenges.

## Author Contributions

**Mohammad Veysi Sheikhrobat:** conceptualization, methodology, validation, formal analysis, writing – original draft. **Sajad Alizadeh:** conceptualization, investigation, funding acquisition, writing – review and editing. **Kamal Shahamiri:** conceptualization, investigation, visualization, writing – review and editing. **Saeed Shahriari:** conceptualization, writing – review and editing, visualization, funding acquisition. **Ahmad Tahmasebi‐Ghorrabi:** supervision, project administration, writing – review and editing, writing – original draft.

## Funding

The authors have nothing to report.

## Ethics Statement

This study was approved by the Ethics Committee of Iran University of Medical Sciences (IUMS). The ethics code: IR.IUMS.REC.1401.845. Informed consent was waived by the Ethics Committee because the study was based exclusively on published literature, policy documents, and secondary publicly available data.

## Consent

The authors have nothing to report.

## Conflicts of Interest

The authors declare no conflicts of interest.

## Transparency Statement

Mohammad Veysi Sheikhrobat affirms that this manuscript is an honest, accurate, and transparent account of the study being reported; that no important aspects of the study have been omitted; and that any discrepancies from the study as planned have been explained.

## Data Availability

The data that support the findings of this study are available from the corresponding author upon reasonable request.
